# Deciphering the interaction surface between the West Nile virus NS3 and NS5 proteins

**DOI:** 10.1099/acmi.0.000675.v3

**Published:** 2024-06-26

**Authors:** Carolin Brand, Brian J. Geiss, Martin Bisaillon

**Affiliations:** 1 Département de Biochimie et de Génomique Fonctionnelle, Université de Sherbrooke, Sherbrooke, QC, Canada; 2 Department of Microbiology, Immunology, and Pathology, Colorado State University, Fort Collins, CO, USA; 3 School of Biomedical Engineering, Colorado State University, Fort Collins, CO, USA

**Keywords:** NS3, NS5, protein–protein interaction, West Nile virus

## Abstract

West Nile virus (WNV) is the most prevalent mosquito-borne virus and the leading cause of viral encephalitis in the continental United States. It belongs to the family *Flaviviridae* which includes other important human pathogens such as dengue virus (DENV), Japanese encephalitis virus (JEV) and Zika viruses (ZIKV). Despite several decades of research, no specific antiviral drugs are available to treat flavivirus infections. The present study characterizes the interaction between the WNV NS3 and NS5 proteins for the purpose of identifying hotspots in the protein-protein interaction which could be targeted for the development of antiviral therapeutics. We previously developed an interaction model *in silico* based on data available in the literature. Here, potential interacting residues on NS3 and NS5 were mutated in a WNV replicon, and seven mutations in the NS3 protein were found to drastically reduce viral replication. In addition to being well conserved among mosquito-borne flaviviruses, these residues are located on the protein’s surface in two clusters which might be interesting new targets for future drug development.

**Impact Statement**

West Nile virus (WNV) is present on all continents except Antarctica, making it the most widespread flavivirus [37,38]. Although approximately 80 % of infections are asymptomatic, and most symptomatic cases are associated with a flu-like illness, WNV can lead to severe illness including neurological symptoms in less than 1 % of infections. Currently, no specific antiviral drug is available, and treatment consists of supportive care to relieve symptoms [15]. Protein–protein interactions have emerged as an interesting target for the development of therapeutic compounds [19,20]. We previously developed a model for the interaction between WNV NS3 and NS5 proteins [27]. Here, we identified residues in a potential interaction interface, and we evaluated the importance of these residues for viral replication. We identify two ‘hotspots’ on the surface of the WNV NS3 protein that are crucial for efficient viral replication, possibly by mediating the interaction between NS3 and NS5, which could be new targets for the development of anti-flaviviral drugs.

## Data Summary

The authors confirm all supporting data, code and protocols have been provided within the article or through supplementary data files. Supplementary data can be found on Figshare: https://doi.org/10.6084/m9.figshare.25847731.v2 [1].

## Introduction

West Nile virus (WNV) is a member of the genus *Flavivirus* (family *Flaviviridae*) and has a positive-sense, ssRNA genome of approximately 11 kb. The genome is capped at the 5′ end but not polyadenylated at the 3′ end, and it contains a single ORF. It is translated into a viral polyprotein which is subsequently processed into ten proteins, namely the structural proteins C, prM and E, as well as the non-structural proteins NS1, NS2A, NS2B, NS3, NS4A, NS4B and NS5 [2,3]. Among the viral proteins, only two exhibit enzymatic functions. NS3 possesses a protease domain and a helicase domain. Its protease activity is involved in polyprotein processing [4]. The helicase domain unwinds the dsRNA replication intermediate [5]. This domain also harbours nucleoside triphosphatase (NTPase) [6] and RNA triphosphatase (RTPase) [7] activities, which are responsible for ATP hydrolysis to drive helicase activity and removal of the γ-phosphate from the newly synthesized viral RNA to form a diphosphorylated RNA during synthesis of the 5′ cap structure, respectively. The NS5 protein also possesses two domains, namely a capping enzyme domain and an RNA-dependent RNA polymerase (RdRp) domain. The capping enzyme domain exhibits guanylyltransferase (GTase) activity which transfers a GMP to the 5′ end of nascent viral RNA [8], and methyltransferase (MTase) activity which methylates the N7 position of the guanine as well as the 2′-O position of the first nucleotide’s ribose to complete the 5′ cap structure [9]. The RdRp domain replicates the viral RNA genome [10]. The two viral enzymes NS3 and NS5 have been shown to interact directly [11,13], and they have been proposed to constitute the main components of the viral replicase complex [14].

Previous efforts to develop antiviral drugs against flaviviruses have mainly focused on inhibiting NS3 or NS5, since they have been well characterized and their enzymatic activities are essential for viral replication. Most potential antiviral compounds that have been identified inhibit enzymatic activity either directly by binding the catalytic site, or they induce a conformational change in the protein by binding an allosteric site [15]. Only three of these compounds, all nucleoside analogue inhibitors of the NS5 RdRp, have reached the clinical stage of drug development [16,18].

An alternative strategy for drug development is the targeting of essential protein–protein interactions. This approach has been studied in various contexts, and protein–protein interactions appear to be promising targets for the development of therapeutics against multiple diseases [19,20]. Regarding the development of specific anti-flaviviral therapeutics, several protein–protein interactions have been proposed and studied as potential drug targets, namely the interaction of NS3 with its essential cofactors NS2B [21,22] and NS4B [23,24], as well as the NS3:NS5 interaction. Mutations that abolish the NS3:NS5 interaction have been shown to inhibit dengue virus (DENV) replication [12,13]. Within the last year, two independent groups have reported small molecules that disrupt the NS3:NS5 interaction via binding to NS5, leading to strong antiviral activity against DENV, Zika viruses (ZIKV) and WNV [25,26].

We have previously developed and presented an interaction model for WNV NS3 and NS5 during positive-strand RNA synthesis [27]. In this model, NS3 and NS5 interact via their helicase and RdRp domain, respectively, and their RNA-binding tunnels are aligned. The dsRNA replication intermediate passes through the helical gate of the NS3 helicase domain, resulting in its separation into two individual strands. The 5′ end of the positive strand gets capped by the NS5 capping enzyme domain. The 3′ end of the negative strand is guided through the RNA-binding tunnel of the NS3 helicase domain and is fed into the NS5 RdRp domain, where it serves as template for the synthesis of a new positive strand of viral RNA [27].

Here, we identified residues in a potential interaction interface between WNV NS3 and NS5 based on the previously described model [27]. Site-directed mutagenesis of a WNV replicon [28] was used to determine the importance of these identified residues for viral replication. Seven residues on the NS3 protein were found to be essential for viral replication and to be clustered in two ‘hotspots’ on the protein surface, suggesting a potential protein interaction surface on NS3.

## Methods

### Interaction model

A model of the WNV NS3:NS5 interaction during positive-strand RNA synthesis was developed and described previously [27].

### WNV replicon and site-directed mutagenesis

A WNV replicon (strain WN 956 D117 3B) [28] was used to study viral replication levels of mutants based on the NS3:NS5 interaction model. A replication-defective WNV replicon, containing a NS5 D668V mutation [29], was used as a negative control. Amino acids that were identified as possibly mediating the NS3:NS5 interaction were substituted with alanine residues in the WNV replicon using QuikChange mutagenesis (Agilent) as previously described [30].

### Luciferase assay

BHK17 cells were grown at 37 °C and 5 % CO_2_ in DMEM (Wisent, 319-015 CL) supplemented with 10 % FBS (Wisent, 080-150) and 1 mM sodium pyruvate (Wisent, 600-110-EL). At 24 h prior to transfection, cells were seeded into 12-well plates at a concentration of 50 000 cells per well. They were then transfected with 250 ng of the replicon expression plasmid using 0.5 µl Lipofectamine 2000 transfection reagent (Invitrogen, 11668019) in opti-MEM (Gibco, 31958070). Luciferase activity was determined 48 h post-transfection by mixing 10 µl of cell lysate (total amount of lysate for each well: 200 µl) with 50 µl of luciferase assay substrate (Promega, E4550) and measuring emitted light with a GloMax 20/20 Luminometer (Promega). Data were collected from at least three independent experiments, each containing technical triplicates. Luciferase activity from the wild-type replicon was fixed at 100 %, and luciferase activity from each mutant was expressed relative to the wild-type. A one-sample t-test with a hypothetical value of 100 was performed for each mutant. Mutants with a *P*-value below 0.05, 0.005, 0.001 and 0.0001 were labelled with one, two, three and four asterisks, respectively.

### Conservation of amino acids among flaviviruses

NS3 and NS5 protein sequences of the WNV replicon were aligned with the corresponding sequences of closely related flaviviruses, namely Japanese encephalitis virus (JEV) (AY303791.1), ZIKV (KU321639.1), DENV1 (KJ189367.1), DENV2 (AY037116.1), DENV3 (AY662691), DENV4 (KF955510.1) and yellow fever virus (YFV) (AF094612.1), using PRALINE multiple sequence alignment with default parameters (available at https://www.ibi.vu.nl/programs/pralinewww/). Conservation scoring was also performed by PRALINE, with 0 being the least conserved alignment position and 10 (*) being the most conserved alignment position.

### Co-immunoprecipitation and western blot

HEK293T cells were grown at 37 °C and 5 % CO_2_ in DMEM (Wisent, 319-015 CL) supplemented with 10 % FBS (Wisent, 080-150). At 24 h prior to transfection, cells were seeded into 6 cm dishes at a concentration of 1000 000 cells per dish. Then, 2.5 µg of pcDNA3.1+ NS3 (with a C-terminal myc tag) and 2.5 µg of pcDNA3.1+ NS5 (with a C-terminal Flag tag) were mixed with 30 µl PEI (Sigma-Aldrich, 408727) in opti-MEM (Gibco, 31958070), incubated for 15 min at room temperature and added to the cells. At 48 h post-transfection, cells were harvested using trypsin, pelleted and stored at −80 °C.

Cells were lysed by adding 300 µl lysis buffer (50 mM Tris/HCl pH 7.5, 150 mM NaCl, 0.1 % NP-40) with cOmplete protease inhibitor (Roche, 11873580001, one tablet per 50 ml) for 10 min at room temperature, followed by 2×5 s sonication at 15 % amplitude (Branson Digital Sonifier). Total protein concentration of the cell lysate was determined using a Bradford assay (Thermo Scientific, 23200). For samples treated with RNase A, 300 µg of total proteins were incubated with 5 µg RNase A (Biotech, RB0473) for 10 min at room temperature. Prior to co-immunoprecipitation (co-IP), 20 µl of anti-DYKDDDDK(Flag) Affinity Gel (Bimake, B23101) was conditioned with 1 ml lysis buffer with protease inhibitor and centrifuged at 2 500 r.p.m. for 5 min at 4 °C. Once the supernatant was removed, cell lysate (300 µg of total proteins) and 1 ml lysis buffer with protease inhibitor were added to the beads and incubated for 4 h at 4 °C on a rotating mixer. Samples were then centrifuged at 2 500 r.p.m. for 5 min at 4 °C, washed with 1 ml lysis buffer without protease inhibitor and centrifuged at 2 500 r.p.m. for 5 min at 4 °C. The wash step was repeated four times. Finally, bound proteins were eluted using 40 µl loading buffer (200 mM Tris/HCl pH 6.8, 40 % glycerol, 4 % SDS, 1.47 M β-mercaptoethanol, bromophenol blue).

A 20 µl aliquot of the eluate was separated using one-dimensional SDS-PAGE at 180 V for approximately 1 h before being transferred to a PVDF blotting membrane (GE, 10600023) at 100V for 75 min. After blocking in 5 % non-fat milk in PBS for 1 h at room temperature, the membranes were incubated with a 1 : 5 000 dilution of primary antibody (NS3-myc: Myc Tag Polyclonal Antibody, Invitrogen, PA1-981; NS5-Flag: Monoclonal Anti-Flag M2 Antibody, Sigma, F1804) in 2.5 % non-fat milk/PBS for 4 h at room temperature, washed three times in TBS-T for 5 min, incubated with a 1 : 10 000 dilution of anti-mouse/rabbit HRP secondary antibody [NS3-myc: Goat Anti-Rabbit IgG H and L (HRP), Abcam, ab205718; NS5-Flag: Anti-mouse IgG HRP-linked Antibody, NEB, 7076] for 1 h at room temperature, and washed twice in TBS-T and once in PBS for 5 min. Finally, ECL substrate (Bio-Rad, 1705061) was used to visualize the target proteins.

### Generation of AlphaFold2

NS3 and NS5 gene sequences from the West Nile Virus Lineage 2 genome (NC_001563) were co-folded using local AlphaFold multimer [31,32] using 8GZR.pdb as template and 20 recycles on a single AMD EPYC 7443 server with two NVIDIA RTX A5000 GPU cards. The model with the highest pLDDT model is presented.

## Results

### Site-directed mutagenesis of residues predicted to mediate the NS3:NS5 interaction

**Fig. 1.**
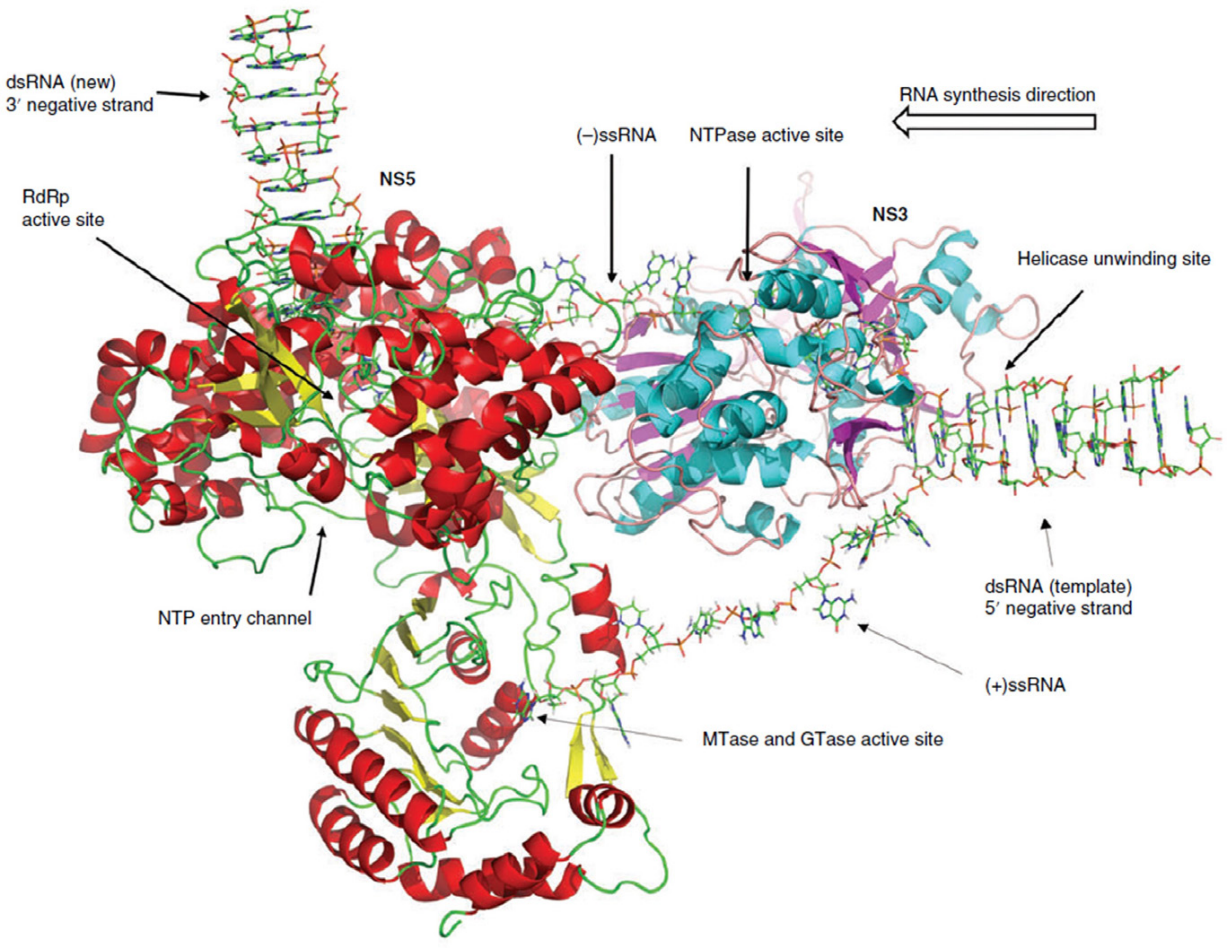
WNV NS3:NS5 interaction model. In the interaction model [27], the incoming dsRNA is unwound by the NS3 helicase. The 5′ end of the positive strand gets capped by the NS5 capping enzyme domain. The 3′ end of the negative strand is guided through the RNA-binding tunnel of the NS3 helicase domain and is fed into the NS5 RdRp domain, where it serves as template for the synthesis of a new positive strand of viral RNA. This figure has been reproduce with permission from John Wiley and Sons.

**Fig. 2.**
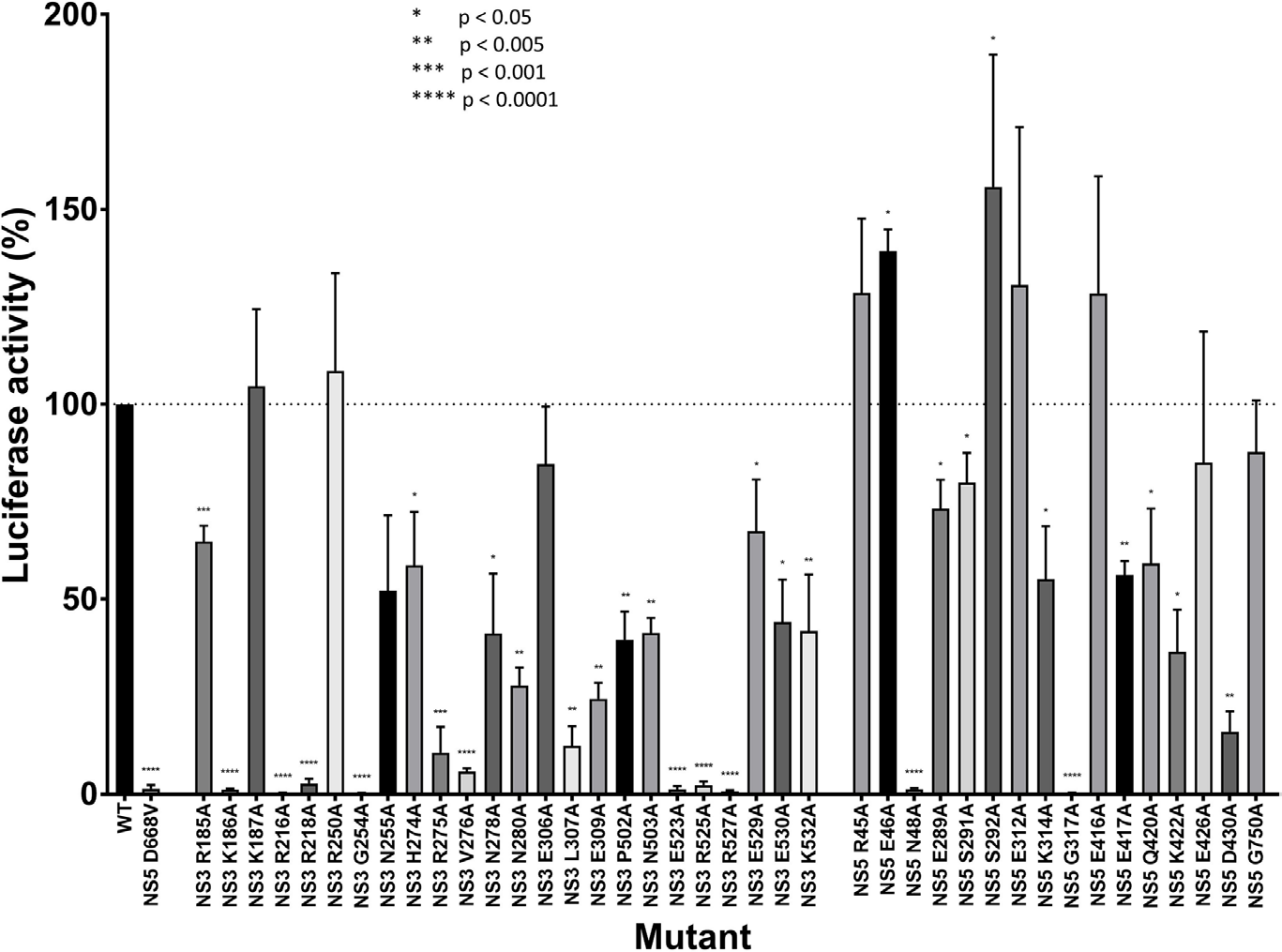
Viral replication levels after alanine substitutions in a WNV replicon. Amino acids in a potential interaction interface between WNV NS3 and NS5 according to the interaction model were replaced by alanine residues in a WNV replicon encoding a luciferase gene. Levels of viral replication were evaluated by measuring luciferase activity in at least three independent experiments, each containing technical triplicates. Luciferase activity from the wild-type replicon was fixed at 100 %, and luciferase activity from each mutant was expressed relative to the wild-type. A one-sample t-test with a hypothetical value of 100 was performed for each mutant. Mutants with a *P*-value below 0.05, 0.005, 0.001 and 0.0001 were labelled with one, two, three and four asterisks, respectively. The following mutations reduced viral replication below 5 % of the WT replicon: NS3 K186A, NS3 R216A, NS3 R218A, NS3 G254A, NS3 E523A, NS3 R525A, NS3 R257A, NS5 N48A and NS5 G317A.

**Fig. 3.**
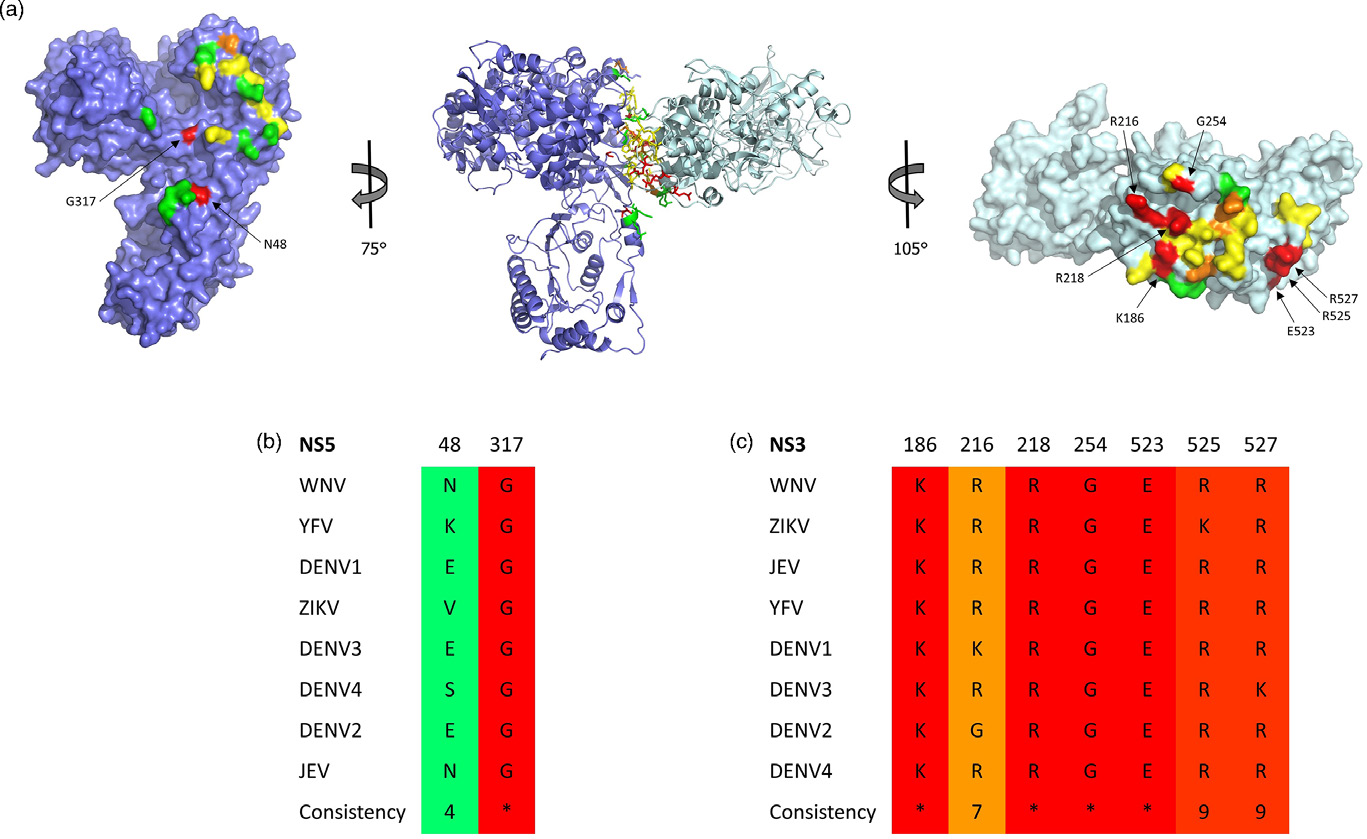
Amino acids whose substitution with alanine reduce replication levels below 5 % of the wild-type replicon. (**a**) The position of amino acids at the potential NS3:NS5 interaction surface chosen for site-directed mutagenesis are shown on the NS3:NS5 interaction model as well as on the individual proteins (left and right). Residues are coloured according to the effect of alanine substitution on viral replication (>80 % of WT is green, 20–80 % of WT is yellow, 5–20 % of WT is orange and <5 % of WT is red). (**b and c**) Conservation of the highlighted amino acids among mosquito-borne flaviviruses was analysed by PRALINE and is represented as ‘Consistency’ which ranges from 0/unconserved to 10(*)/conserved.

In this study, we identified residues which might be involved in the WNV NS3:NS5 interaction based on our previously developed interaction model [27]. In this model, NS3 and NS5 interact via their helicase and RdRp domains, respectively (Fig. 1). Numerous residues that appeared to be directly interacting with the facing protein as well as residues pointing towards the inter-protein space were hypothesized to mediate the potential NS3:NS5 interaction. These include NS3 amino acids R185, K186, K187, R216, R218, R250, G254, N255, H274, R275, V276, N278, N280, E306, L307, E309, P502, N503, E523, R525, R527, E529, E530 and K532, as well as the NS5 amino acids R45, E46, N48, E289, S291, S292, E312, K314, G317, E416, E417, Q420, K422, E426, D430 and G750. To evaluate the importance of these residues, site-directed mutagenesis was performed in a WNV replicon, and replication levels of mutants were compared to the wild-type (Fig. 2). A replication-defective WNV replicon, containing an NS5 D668V mutation [29], was also used as a negative control. Our results demonstrate that some mutations had little or no effect, and some even increased viral replication. Interestingly, several alanine substitutions were found to reduce viral replication below 5 % of the wild-type (WT) replicon, namely NS3 K186A, NS3 R216A, NS3 R218A, NS3 G254A, NS3 E523A, NS3 R525A, NS3 R257A, NS5 N48A and NS5 G317A, suggesting that residues at these positions are critical for efficient viral replication. Next, we investigated whether these residues are conserved among related flaviviruses (Fig. 3). Only two of these nine residues have a conservation score below 9 (PRALINE conservation score, 0 being least conserved and 10 being most conserved) when comparing WNV, ZIKV, JEV, YFV and all four serotypes of DENV, which further suggests an important role for these residues.

### Co-immunoprecipitation of mutant NS3 with wild-type NS5

**Fig. 4.**
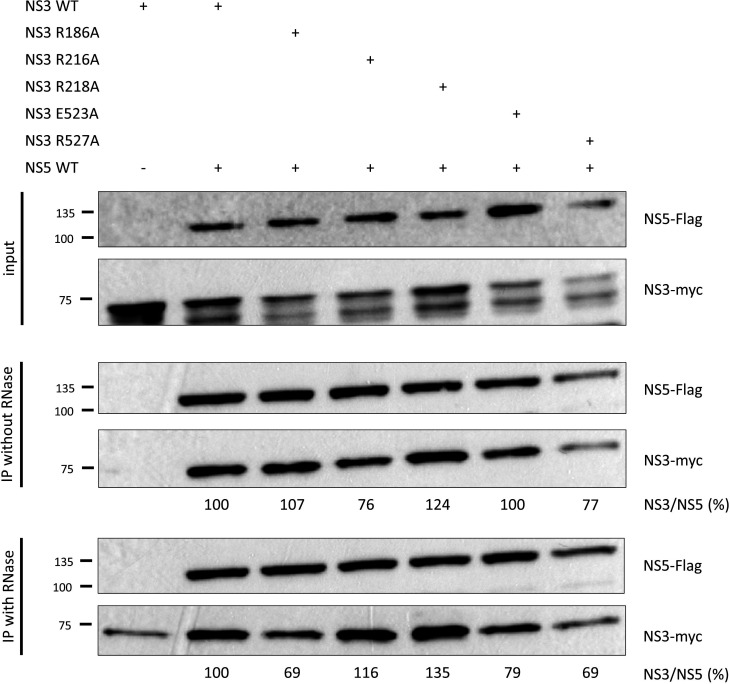
Co-immunoprecipitation of NS3 mutants with wild-type NS5. Cells were co-transfected with WT Flag-tagged NS5 and either WT or mutant myc-tagged NS3. NS5 was immunoprecipitated, and co-precipitated NS3 was revealed by western blotting. The NS3/NS5 percentage ratio was calculated and is shown under each blot.

We next examined whether the observed loss of viral replication is due to a loss of interaction between NS3 and NS5 by co-IP of mutant NS3 proteins with wild-type NS5 (Fig. 4). Co-IP was observed for all mutants, with and without RNase treatment. RNase treatment was used to rule out indirect interaction via RNA, since both proteins are known to bind RNA. No drastic loss of interaction between the two proteins was observed, although the mutations R186A and E523A on NS3 had a modest negative effect on the interaction with NS5. At first sight, these results may suggest that the observed reduction in viral replication is not caused by an impaired NS3:NS5 interaction. However, it is very likely that multiple pairs of interactions are present between NS3 and NS5, and breaking one pair would be expected to reduce interaction affinity but not completely ablate the interaction. Alternatively, these results might suggest that the formation of the NS3:NS5 complex is mediated by different pairs of interacting residues during different stages of viral replication. The previously described mutations may prevent NS3 and NS5 from interacting in a specific way at one step of viral replication, resulting in loss of viral replication, but other ways of interacting during other phases of viral replication may be unaffected, leading to sustained interaction and co-IP *in vitro*.

### Comparison with the recently published cryo-EM structure of the DENV replicase complex

**Fig. 5.**
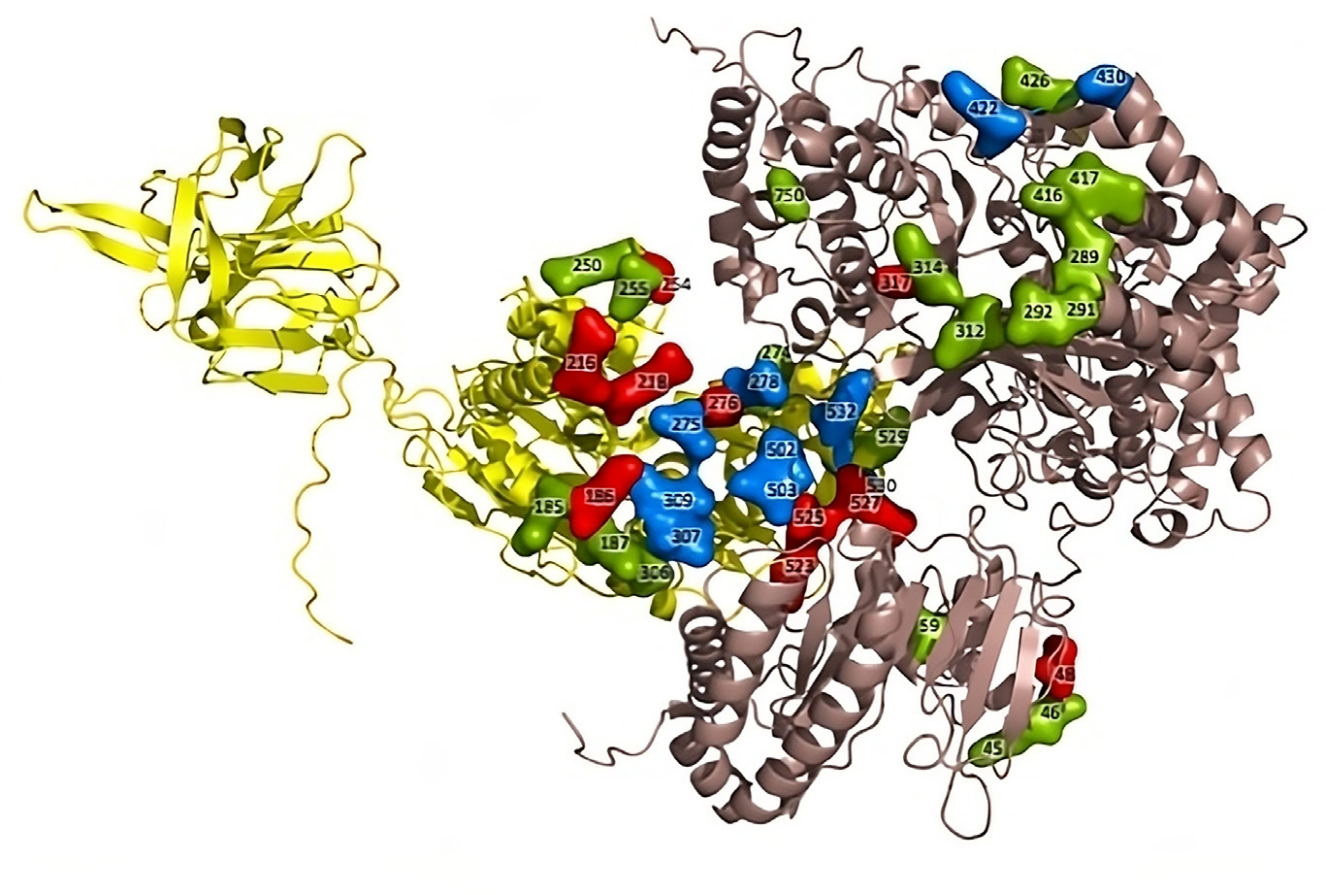
Alphafold2 model of the WNV NS3:NS5 complex based on the DENV replicase cryo-EM structure. The recently published cryo-EM structure of the DENV replicase complex (8GZR) was used as a guide to build an Alphafold2 model of the WNV NS3:NS5 complex, with NS3 and NS5 being shown in yellow and salmon, respectively. The above-mentioned mutations were mapped onto this new model and coloured according to their effect on viral replication, with red being less than 10 % of the WT, blue being 10–50 % of the WT and green being more than 50 % of the WT. Residue numbers are noted.

Recently, during the review process of this paper, cryo-electron microscopy (cryo-EM) structures of DENV RNA replicase complexes were published [33]. Therefore, we built an Alphafold2 model [31,32] of the WNV NS3:NS5 complex using the DENV structure (8GZR) as a guide and mapped the previously described mutations onto this new model (Fig. 5). Based on this, three critical residues of NS3 are putative interaction points with NS5, namely E523, R525 and R527.

## Discussion

In the present study, we experimentally tested our previously published interaction model of WNV NS3 and NS5 during positive-strand RNA synthesis [27] by identifying residues in a potential interaction interface. These residues were then substituted with alanine in a WNV replicon, and the effects of these mutations on viral replication were evaluated. Nine substitutions were found to reduce viral replication below 5 % of the wild-type replicon, suggesting that these residues are essential for efficient viral replication. Moreover, when aligning protein sequences from several pathogenic mosquito-borne flaviviruses, we discovered that seven of these nine residues are highly conserved among WNV, ZIKV, JEV, YFV and DENV, which further highlights their importance. Five of these alanine substitutions on NS3 were investigated for their ability to directly interact with wild-type NS5, and only two of them showed a limited loss of interaction. This suggests that single point mutations may not be sufficient to disrupt the NS3:NS5 interaction. Alternatively, they may not be involved in the NS3:NS5 interaction, but rather have some other function (e.g. structure, allostery).

Previous studies on DENV NS3 and NS5 have identified one residue of each protein, namely NS3 N570 [13] and NS5 K330 [12], to be involved in the NS3:NS5 interaction. The corresponding residues were mutated in our WNV replicon, and viral replication of the mutants was evaluated (Fig. S1A, available in the online version of this article). Reduction of viral replication below 5 % of the wild-type replicon was observed for the NS5 K332A mutant (WNV numbering). However, the NS3 N571A (WNV numbering) substitution had no significant effect on viral replication. This may suggest that, despite both residues being well conserved among mosquito-borne flaviviruses (Fig. S1B), only the role of NS5 K332, but not that of NS3 N571, in the NS3:NS5 interaction is conserved among flaviviruses.

The current study identified two ‘hotspots’ on the surface of the WNV NS3 protein that are crucial for efficient viral replication, possibly by mediating the interaction between NS3 and NS5, which could be alternative targets for the development of anti-flaviviral drugs. To better identify NS3:NS5 interactions and confirm that the two ‘hotspots’ on NS3 identified here mediate the interaction with NS5, cross-link MS could be used. Alternatively, cryo-EM could be used to better understand how the NS3:NS5 complex is assembled. This is important since the Alphafold2 model of the WNV NS3:NS5 complex generated using the DENV structure (8GZR) as a guide is structurally different from our initial model. Moreover, it has become increasingly clear that many RNA viruses that form replication compartments on cellular membranes organize their replication proteins into complex pore structures [34,36], which is likely for flaviviruses as well, although this has not been shown yet. It is highly likely that the DENV replicase cryo-EM structure simply represents a monomer in a larger oligomeric complex that forms a pore structure, and there is a higher order NS3:NS5 structure that is not yet known. Interestingly, there are quite a few deleterious surface mutations in NS3 and NS5 (replicon activity below 10 %) that are in non-contact points in the cryo-EM model, which could be representative of interaction points between different subunits of an oligomeric pore structure. Elucidating the precise nature of NS3:NS5 interactions and the architecture of this complex structure could not only shed light on the complex virology of flaviviruses but also open new avenues for the development of innovative and more effective antiviral therapies.

## supplementary material

10.1099/acmi.0.000675.v3 Fig. S1.

## Abbreviations

co-IP: co-immunoprecipitation
cryo-EM: cryo electron microscopy
DENV: dengue virus
GTase: guanylyltransferase
MTase: methyltransferase
NTPase: nucleoside triphosphatase
RdRp: RNA-dependent RNA polymerase
RTPase: RNA triphosphatase
WNV: West Nile virus
YFV: yellow fever virus
ZIKV: Zika virus

## References

[1] Brand C, Geiss BJ, Bisaillon M (2024). *Figshare*.

[2] Chambers TJ, Hahn CS, Galler R, Rice CM (1990). Flavivirus genome organization, expression, and replication. Annu Rev Microbiol.

[3] Lindenbach BD, Thiel H-J, Rice CM, Knipe DM, Howley PM (2007). In Fields Virology.

[4] Wengler G, Czaya G, Färber PM, Hegemann JH (1991). In vitro synthesis of West Nile virus proteins indicates that the amino-terminal segment of the NS3 protein contains the active centre of the protease which cleaves the viral polyprotein after multiple basic amino acids. J Gen Virol.

[5] Du Pont KE, Sexton NR, McCullagh M, Ebel GD, Geiss BJ (2020). A hyperactive Kunjin Virus NS3 helicase mutant demonstrates increased dissemination and mortality in mosquitoes. J Virol.

[6] Wengler G, Wengler G (1991). The carboxy-terminal part of the NS 3 protein of the West Nile flavivirus can be isolated as a soluble protein after proteolytic cleavage and represents an RNA-stimulated NTPase. Virology.

[7] Wengler G, Wengler G (1993). The NS 3 nonstructural protein of flaviviruses contains an RNA triphosphatase activity. Virology.

[8] Issur M, Geiss BJ, Bougie I, Picard-Jean F, Despins S (2009). The flavivirus NS5 protein is a true RNA guanylyltransferase that catalyzes a two-step reaction to form the RNA cap structure. RNA.

[9] Ray D, Shah A, Tilgner M, Guo Y, Zhao Y (2006). West Nile virus 5’-cap structure is formed by sequential guanine N-7 and ribose 2’-O methylations by nonstructural protein 5. J Virol.

[10] Guyatt KJ, Westaway EG, Khromykh AA (2001). Expression and purification of enzymatically active recombinant RNA-dependent RNA polymerase (NS5) of the flavivirus Kunjin. J Virol Methods.

[11] Kapoor M, Zhang L, Ramachandra M, Kusukawa J, Ebner KE (1995). Association between NS3 and NS5 proteins of dengue virus type 2 in the putative RNA replicase is linked to differential phosphorylation of NS5. J Biol Chem.

[12] Zou G, Chen Y-L, Dong H, Lim CC, Yap LJ (2011). Functional analysis of two cavities in flavivirus NS5 polymerase. J Biol Chem.

[13] Tay MYF, Saw WG, Zhao Y, Chan KWK, Singh D (2015). The C-terminal 50 amino acid residues of dengue NS3 protein are important for NS3-NS5 interaction and viral replication. J Biol Chem.

[14] van den Elsen K, Quek JP, Luo D (2021). Molecular insights into the flavivirus replication complex. Viruses.

[15] Qian X, Qi Z (2022). Mosquito-borne flaviviruses and current therapeutic advances. Viruses.

[16] ClinicalTrials.gov (2023). A study of balapiravir in patients with dengue virus infection. https://clinicaltrials.gov/ct2/show/NCT01096576.

[17] ClinicalTrials.gov (2023). A phase 1 study to evaluate the safety, tolerability and pharmacokinetics of BCX4430. https://clinicaltrials.gov/ct2/show/NCT02319772.

[18] ClinicalTrials.gov (2023). Study of AT-752 in healthy subjects. https://clinicaltrials.gov/ct2/show/NCT04722627.

[19] Celegato M, Messa L, Goracci L, Mercorelli B, Bertagnin C (2020). A novel small-molecule inhibitor of the human papillomavirus E6-p53 interaction that reactivates p53 function and blocks cancer cells growth. Cancer Lett.

[20] Marcello A, Loregian A, Cross A, Marsden H, Hirst TR (1994). Specific inhibition of herpes virus replication by receptor-mediated entry of an antiviral peptide linked to *Escherichia coli* enterotoxin B subunit. Proc Natl Acad Sci U S A.

[21] Li Z, Brecher M, Deng Y-Q, Zhang J, Sakamuru S (2017). Existing drugs as broad-spectrum and potent inhibitors for Zika virus by targeting NS2B-NS3 interaction. Cell Res.

[22] Yao Y, Huo T, Lin Y-L, Nie S, Wu F (2019). Discovery, X-ray crystallography and antiviral activity of allosteric inhibitors of flavivirus NS2B-NS3 protease. J Am Chem Soc.

[23] Kaptein SJF, Goethals O, Kiemel D, Marchand A, Kesteleyn B (2021). A pan-serotype dengue virus inhibitor targeting the NS3-NS4B interaction. Nature.

[24] Goethals O, Kaptein SJF, Kesteleyn B, Bonfanti J-F, Van Wesenbeeck L (2023). Blocking NS3-NS4B interaction inhibits dengue virus in non-human primates. Nature.

[25] Yang SNY, Maher B, Wang C, Wagstaff KM, Fraser JE (2022). High throughput screening targeting the dengue NS3-NS5 interface identifies antivirals against dengue, zika and West Nile viruses. Cells.

[26] Celegato M, Sturlese M, Vasconcelos Costa V, Trevisan M, Lallo Dias AS (2023). Small-molecule inhibitor of flaviviral NS3-NS5 interaction with broad-spectrum activity and efficacy *in vivo*. mBio.

[27] Brand C, Bisaillon M, Geiss BJ (2017). Organization of the flavivirus RNA replicase complex. Wiley Interdiscip Rev RNA.

[28] Pierson TC, Sánchez MD, Puffer BA, Ahmed AA, Geiss BJ (2006). A rapid and quantitative assay for measuring antibody-mediated neutralization of West Nile virus infection. Virology.

[29] Gullberg RC, Jordan Steel J, Moon SL, Soltani E, Geiss BJ (2015). Oxidative stress influences positive strand RNA virus genome synthesis and capping. Virology.

[30] Du Pont KE, Davidson RB, McCullagh M, Geiss BJ (2020). Motif V regulates energy transduction between the flavivirus NS3 ATPase and RNA-binding cleft. J Biol Chem.

[31] Jumper J, Evans R, Pritzel A, Green T, Figurnov M (2021). Highly accurate protein structure prediction with AlphaFold. Nature.

[32] Evans R, O’Neill M, Pritzel A, Antropova N, Senior A (2022). Protein complex prediction with AlphaFold-Multimer. bioRxiv.

[33] Osawa T, Aoki M, Ehara H, Sekine S-I (2023). Structures of dengue virus RNA replicase complexes. Mol Cell.

[34] Unchwaniwala N, Zhan H, den Boon JA, Ahlquist P (2021). Cryo-electron microscopy of nodavirus RNA replication organelles illuminates positive-strand RNA virus genome replication. Curr Opin Virol.

[35] Tan YB, Chmielewski D, Law MCY, Zhang K, He Y (2022). Molecular architecture of the Chikungunya virus replication complex. Sci Adv.

[36] Jones R, Hons M, Rabah N, Zamarreño N, Arranz R (2023). Structural basis and dynamics of Chikungunya alphavirus RNA capping by nsP1 capping pores. Proc Natl Acad Sci U S A.

[37] Kramer LD, Ciota AT, Kilpatrick AM (2019). Introduction, spread, and establishment of West Nile virus in the Americas. J Med Entomol.

[38] Hayes EB, Komar N, Nasci RS, Montgomery SP, O’Leary DR (2005). Epidemiology and transmission dynamics of West Nile virus disease. Emerg Infect Dis.

