# Supplementary material for: Deciphering the interaction surface between the West Nile virus NS3 and NS5 proteins
Source: Access Microbiol. 2024 Jun 26;6(6):000675.v3. doi: 10.1099/acmi.0.000675.v3 (PMC11261718; doi:10.1099/acmi.0.000675.v3)
Supplement: Fig. S1. [file acmi-6-00675-s001.pdf]

A

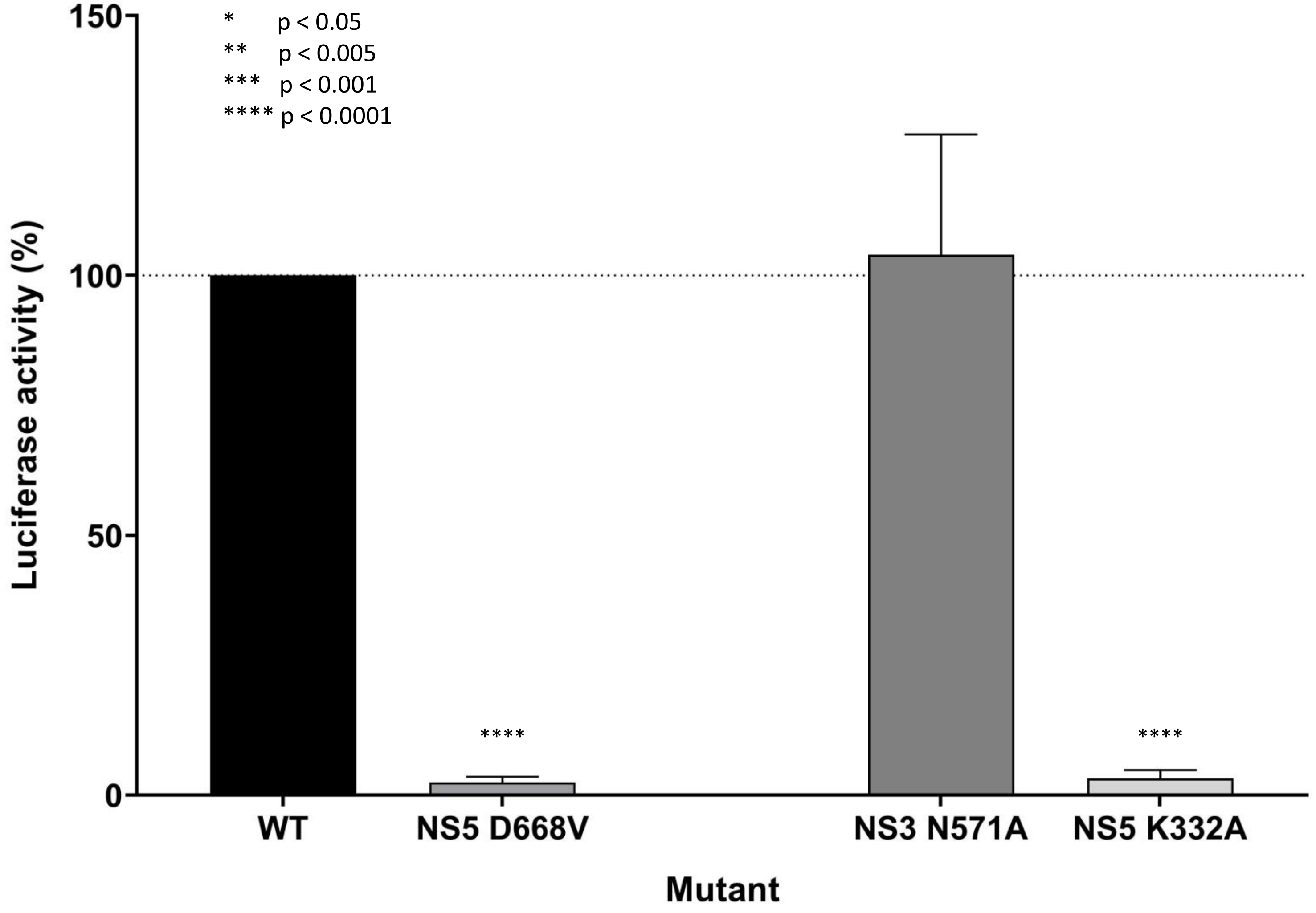

B

	NS3 N571	NS5 K332
WNV	N	K
YFV	H	Y
DENV1	N	K
ZIKV	N	K
DENV3	N	K
DENV4	N	K
DENV2	N	K
JEV	N	K
Consistency	8	8

Figure S1. Viral replication levels after alanine substitutions of residues previously shown to be involved in the DENV NS3-NS5 interaction. DENV NS3 N570 and DENV NS5 K330 have been demonstrated to be critical for the DENV NS3-NS5 interaction [17,18]. (A) The corresponding residues in the WNV replicon were substituted by alanine, and levels of viral replication were evaluated by measuring luciferase activity in four independent experiments, each containing triplicates. (B) Conservation of amino acids (WNV numbering) among mosquito-borne Flaviviruses was analyzed by PRALINE.
